# Short lasting activity-related headaches with sudden onset in children: a case-based reasoning on classification and diagnosis

**DOI:** 10.1186/1129-2377-14-3

**Published:** 2013-01-25

**Authors:** Irene Toldo, Debora De Carlo, Rodica Mardari, Luca De Palma, Michela Gatta, Barbara Bolzonella, Margherita Nosadini, Luca Bartolini, Stefano Sartori, Pier Antonio Battistella

**Affiliations:** 1 Juvenile Headache Centre, Department of Woman and Child Health, University of Padua, Via Giustiniani, 3, 35128, Padova, Italy; 2 Institute of Neuroradiology, Padua Hospital, Padua, Italy

**Keywords:** Children, Cough headache, Exertional headache, Secondary headaches, Chiari 1 malformation

## Abstract

**Background:**

Short lasting headaches related to activity or cough are rare, particularly in childhood, and can be difficult to diagnose, especially in young children who are not able to describe their symptoms. In the literature there are few data on this topic in adults and the paediatric cases reported are even more rare.

**Findings:**

We present the clinical history of a 7-year-old child and a 3-year-old child both diagnosed as having activity-related headaches, characterized by sudden onset of short lasting (few seconds) attacks, that were triggered by cough or exercise. There were no accompanying symptoms and the neurological examination was normal in both cases. Brain magnetic resonance imaging showed, in the first case, a cerebellar pilocytic astrocytoma and, in the second case, a Chiari 1 malformation. Both cases received an early diagnosis, were surgically treated and had a good prognosis at follow-up.

**Conclusions:**

When headache has a recent onset, it presents suddenly, and it is triggered by strain, even with normal neurological examination, neuroimaging is mandatory in order to exclude secondary headaches, especially in children.

## Introduction

Primary cough headache and primary exertional headache, included respectively in paragraph 4.2 and 4.3 of the current International Classification of Headache Disorders (ICHD-II, 2004) [1], are rare in adults [2-11], but especially in children [4]. Recently some Authors have reported a higher prevalence of primary exertional headache in adolescents [6-8]. Differential diagnosis requires consideration of secondary headaches attributable to:

a) Chiari 1 malformation (CM1) (7.7, ICHD-II) [1];

b) High cerebrospinal fluid pressure (7.1, ICHD-II) [1];

c) Intracranial neoplasm (7.4, ICHD-II) [1].

We describe the history of two children with activity-related headaches in which a diagnosis of secondary headache was made. The clinical studies were conducted according to routinely adopted protocols approved from the local ethics committee. We discuss the possible differential diagnosis on the basis of the headache pattern and the clinical issues that raised a suspicion for secondary life-threatening headaches in our cases.

## Findings

### Case 1

**Figure 1:**
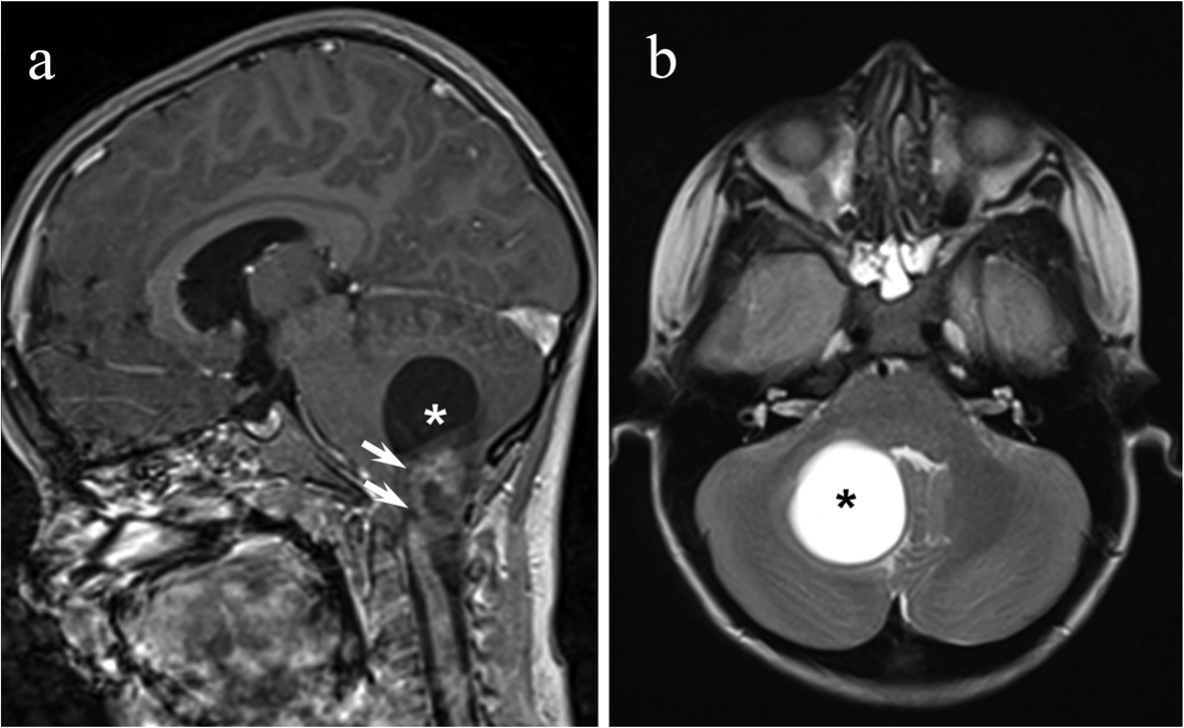
Sagittal contrast-enhanced T1-weighted (a) and axial T2-weighted (b) images showing cystic cerebellar mass (asterisk) with a large dyshomogeneous enhancing nodule (white arrows) arising from the left cerebellar hemisphere.

A 7-year-old boy, two days after a minor trauma, started to complain attacks of headache “as a pinch/stab” at the nuchal region, lasting seconds, twice a day, without other associated symptoms. The mother suffered for migraine without aura. His past personal history was unremarkable. After two days he had an isolated episode of vomiting without headache; then he complained headache only during coughing or exercise, sometimes associated with dizziness and mild unsteady gait. He had a stabbing and bilateral pain at the occipital region; the headache was moderate in intensity, had sudden onset, and lasted few seconds. Apart from the headache attacks, the child was asymptomatic. He presented to our Hospital after two months for a single episode of paroxysmal torticollis lasting about two hours; at admission neurological examination and fundus oculi were normal. Considering the atypical headache pattern, the episode of vomiting without headache and the paroxysmal torticollis, he underwent a brain magnetic resonance imaging (MRI) that showed a cerebellar expansive lesion (Figure 1) and a triventricular hydrocephalus. The spinal MRI was negative. The cerebellar lesion was surgically removed with complete exeresis; the histopathological examination was consistent with a pilocytic astrocytoma (grade 1, World Health Organization). At a 2-year follow-up the neurological examination was normal and the child was completely asymptomatic.

### Case 2

A 3-year-old child complained headache attacks since the age of 18 months, 3 weeks after a minor head trauma. He had attacks of sudden short lasting (few seconds) headache, only during cough or exertion (as lifting a weight), at the occipital region, without nausea or vomiting. The father suffered from migraine without aura.

**Figure 2:**
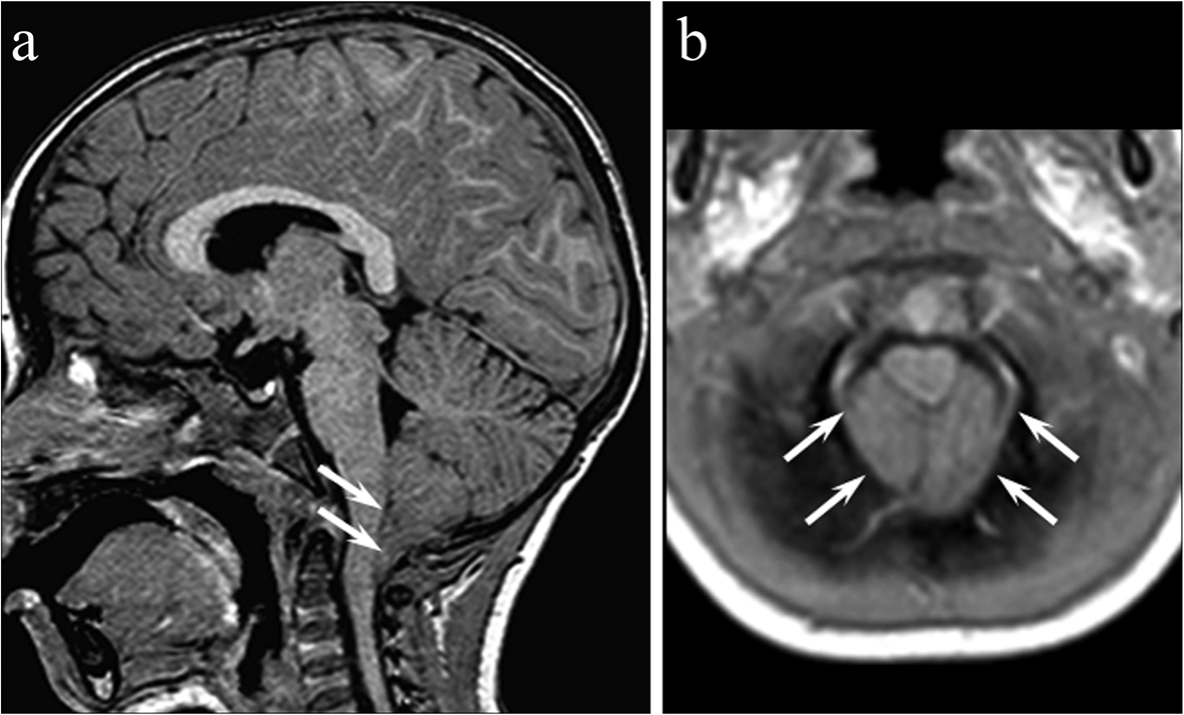
Sagittal contrast-enhanced T1-weighted (a) showing a caudal protrusion (12 mm) of the elongated triangular-shaped tonsils below the foramen magnum (white arrows). Axial T1-weighted image (b) confirms cerebellar tonsils filling the foramen magnum and effacing the cysterna magna (white arrows).

His past personal history was apparently unremarkable but when parents were specifically asked if there were signs or symptoms of CM1, they reported some episodes of dysphagia to liquids, falls and numbness at fours limbs. Fundus oculi and neurological examination (24 months) were normal. The child was visited at our Hospital at the age of 25 months, because of a worsening of headache frequency in the last 2 months; the neurological examination was normal. A brain MRI (26 months) showed the herniation of the cerebellar tonsils (12 mm) through the foramen magnum until the second cervical vertebra, consistent with a CM1 (Figure 2). Spinal MRI showed a compression of the spinal cord with abnormal signal on T2-weighted images at the level of the second cervical vertebra, without syringomyelia. As the child had a symptomatic CM1 and also signal abnormalities in the upper cervicalmedulla, a surgical decompression of the posterior fossa was performed (27 months). One month later (28 months) headache and dysphagia to liquids disappeared and the other neurological symptoms (falls and numbness at fours limbs) significantly improved; at this age cine phase-contrast MRI showed normal aqueductal cerebrospinal fluid flow and the neurological examination was normal.

## Discussion

Activity-related headaches can be brought on by Valsalva maneuvers (“cough headache”) or prolonged exercise (“exertional headache”). These headaches account overall for 1-2% of the consultations due to headache in a general neurological Department for adults [9], while in children they are very rare. These entities are a challenging diagnostic problem as they can be primary or secondary and as their etiologies differ depending on the headache pattern [9].

Primary cough headache (PCH) is considered to be a rare condition, accounting for 0.4% of all headaches consulting a Neurology Department, and predominantly affects male patients older than 40 years of age [4,5,9-11]; its pathophysiology is unknown [4,5,9-11]. PCH is a sudden-onset headache that usually lasts from 1 second to 30 minutes, tends to be bilateral and posterior, is brought on by and occurring only in association with coughing, straining and/or Valsalva maneuver, and it responds to indomethacin [4,5,9-14]. A craniocervical MRI study is mandatory to rule out posterior fossa lesions [5,9]; in fact cough headache can be symptomatic in about 40% of cases and the large majority of them are due to CM1 [4,5,9-11], as in case 2 of the present study. Other reported causes of symptomatic cough headache include carotid or vertebrobasilar diseases and cerebral aneurysms. Differently from PCH, secondary cough headache begins earlier (average 40 vs 60 years), is located posteriorly, lasts longer (years vs months), is associated with posterior fossa signs or symptoms, and does not respond to indomethacin [9,10].

Among the 72 cases with activity-related headaches described by Pascual *et al.*[5] there were 30 patients with cough headache (13 primary, 17 secondary) and 28 cases with exertional headache (16 primary; 12 secondary); the few cases (the exact number was not specified) under the age of 18 years had secondary cough headache or primary exertional headache. Age at onset of secondary cough headache (mean 39 ± 14, range 15 - 63) was significantly lower than for PCH (mean 67 ± 11, range 44 - 81). All cases with secondary cough headache had a CM1, and most of them (14/17) complained posterior fossa symptoms or signs apart from headache; the three patients having isolated headache developed posterior fossa symptoms or signs after an interval between 1 to 5 years [5].

In our two cases we investigated whether the pattern of headache met the diagnostic criteria of ICHD-II [1]. In presence of cough headache, that both our patients presented, the criteria A-B-C of PCH (4.2, ICHD-II) [1] are satisfied, but the diagnosis requires that headache is not attributed to an another disorder. In fact in our cases, by mean of brain MRI showing respectively a cerebellar neoplasm (case 1) and a CM1 (case 2), a diagnosis of secondary cough headache could be made. Considering other possible encodings, case 1 met all the criteria for headache attributed to intracranial neoplasm (7.4.2, ICHD-II) [1]. Moreover the child had an isolated episode of paroxysmal torticollis, lasting about two hours, that was a strong element of suspicion for craniocervical pathology. Case 2 fulfilled the criteria A, B and D for headache attributed to CM1 (7.7, ICHD-II) [1], while criteria C was only partially satisfied. This is probably due to the fact that the young age of child made difficult to refer and therefore to recognize transient visual or oto-neurological symptoms [15-19]; moreover the follow-up was not long enough to establish if the patient will develop posterior fossa symptoms or signs within 5 years, as reported in the study by Pascual *et al. *[5].

In our two cases headache was triggered not only by cough but also by exertion, therefore we considered another diagnostic category, exertional headache.

Primary exertional headache (PEH) can present in adolescence [6-8], last more than 5 minutes and is often associated with disautonomic symptoms. Among 72 cases with activity-related headaches, primary exertional headache (age: 24±11, 10-48) began significantly earlier than PCH and secondary exertional headache [5]. On first occurrence of this headache type, it is mandatory to exclude subarachnoid haemorrhage and arterial dissection [6-8,12,14].

While in PCH the headache can be triggered by exercise or by coughing, in the PEH pain occur only during or after physical exertion [1,6-8]. Therefore in our cases headache pattern did not meet the criteria for PEH because in both cases headache was triggered by exertion and coughing, and in case 1 the duration of pain was much lower than 5 minutes.

A type of headache where the pain is described as a pinch or a stab and it is short lasting, as in our cases, is primary stabbing headache (4.1, ICHD-II) [1]. It is characterized by transient and localised stabs of pain, single or serial, that occur spontaneously in the absence of organic disease of underlying structures. Case 1 met all the criteria but one, in which the pain is felt in the distribution of the first division of the trigeminal nerve.

Considering the duration of headache in our two cases, also short lasting headaches can be included in the differential diagnosis, in particular for case 1 that initially had short lasting attacks not triggered by Valsalva maneuver.

Short lasting headaches include short-lasting unilateral neuralgiform headache attacks with conjunctival injection and tearing (SUNCT), primary stabbing headache, cluster headache, paroxysmal hemicrania, and neck-tongue syndrome [1,20-25].

SUNCT is classified among primary headaches and it is characterized by short-lasting attacks (from 5 seconds to 4 minutes) of unilateral pain that are much briefer than those seen in any other trigeminal autonomic cephalalgias and very often accompanied by prominent lacrimation and redness of the ipsilateral eye (3.3, ICHD-II) [1]. SUNCT is a very rare syndrome, particularly in childhood [20-22].

Primary stabbing “ice-pick” headache is a primary headache syndrome characterized by transient, sharp, stabbing pains that occur within a small area of the scalp for seconds [23]. The pain tends to occur in the distribution of the first division of the trigeminal nerve, including the orbital, temporal, or parietal regions [23]. Its prevalence in children is estimated at 3-5% and it usually appears by age 10 years [23]. In a large sample of children affected, this type of headache usually was not associated with other primary headache syndromes [24].

Cluster headache (3.1, ICHD-II) [1], that affects adults, consists of strictly unilateral pain attacks, more frequent (1-8 times a day), longer (15-180 minutes) and associated with ipsilateral autonomic symptoms [25]; therefore it can be excluded in our cases. The same applies to paroxysmal hemicrania (3.2, ICHD-II) [1] that is a rare condition, particular in children, similar to cluster headache but with shorter duration (2-30 minutes) and higher frequency (> 5 times a day).

Neck-tongue syndrome (13.9, ICHD-II) [1] consists of a sudden onset of pain in the occipital region or upper neck, associated with abnormal sensation in the same side of the tongue; in our cases one of the three criteria (pain is commonly precipitated by sudden turning of the head) was not satisfied.

In the literature there are no reports on cough headache in children younger than 10 years. In our cases, the clinical elements of suspicion for a secondary headache were the pain caused by the Valsalva maneuver (strain/cough) for both and, in case 1, the recent onset of headache, the nuchal site of pain, an episode of vomiting without headache and stiff neck, while in case 2 the low age of onset (3 years). Our cases demonstrate that midline lesions in young children do not lateralize well on neurological examination. Therefore, recurrent or progressive headache without the other associated features of migraine should be alarming enough to obtain neuroimaging.

In adults almost half of cases (42%) with activity-related headaches had intracranial lesions, and symptomatic cases (57%) prevailed among subjects with cough headaches [5]. This also can be applied to children in which the prevalence, not estimated, of primary cough and exertional headaches is likely to be rarer than in adults.

The association with strain or a cough is therefore an important clinical issue, which should always be investigated and that can be a sign of alarm for secondary headaches, especially in children.

When headache has a recent onset, it presents suddenly, it is triggered by strain, even with normal neurological examination, neuroimaging is mandatory in order to exclude secondary headaches, particularly in children. An early clinical diagnosis allowed in our cases a good control of underlying disease.

### Consent

Written informed consent was obtained from the patients’ parents for the publication of the cases report and any accompanying images. A copy of the written consent is available for review by the Editor-in-Chief of this journal.

### Ethical approval

The clinical management of the patients reported in this paper was conform to the indications provided by our institutional review board.

## Abbreviations

ICHD: International Classification of Headache Disorders;CM1: Chiari 1 malformation;MRI: Magnetic resonance imaging;PCH: Primary cough headache;PEH: Primary exertional headache;SUNCT: Short-lasting unilateral neuralgiform headache attacks with conjunctival injection and tearing

## Competing interests

The authors declare no potential conflicts of interests with respect to the authorship and/or publication of this article.

## Authors’ contributions

IT, DDC, LDP, RM: have made substantial contributions to conception and design, acquisition of data, analysis and interpretation of data; MG, BB, MN, LB: have been involved in drafting the manuscript; IT, SS, PAB: were involved in revising the manuscript critically for important intellectual content and have given final approval of the version to be published. All authors read and approved the final manuscript.
